# Autocatalytic sets and boundaries

**DOI:** 10.1186/s13322-014-0006-2

**Published:** 2015-02-12

**Authors:** Wim Hordijk, Mike Steel

**Affiliations:** SmartAnalytiX.com, Lausanne, Switzerland; Allan Wilson Centre, University of Canterbury, Christchurch, New Zealand

**Keywords:** Autocatalytic sets, Origin of life, Boundaries

## Abstract

Autopoietic systems, chemotons, and autogens are models that aim to explain (the emergence of) life as a functionally closed and self-sustaining system. An essential element in these models is the notion of a boundary containing, maintaining, and being generated by an internal reaction network. The more general concept of collectively autocatalytic sets, formalized as RAF theory, does not explicitly include this notion of a boundary. Here, we argue that (1) the notion of a boundary can also be incorporated in the formal RAF framework, (2) this provides a mechanism for the emergence of higher-level autocatalytic sets, (3) this satisfies a necessary condition for the evolvability of autocatalytic sets, and (4) this enables the RAF framework to formally represent and analyze (at least in part) the other models. We suggest that RAF theory might thus provide a basis for a unifying formal framework for the further development and study of such models.

**Graphical abstract:**
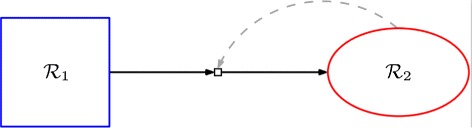
The emergence of an autocatalytic (super)set of autocatalytic (sub)sets.

The emergence of an autocatalytic (super)set of autocatalytic (sub)sets.

## Report

### Introduction

The theory of *autopoietic systems* [1-3] and the *chemoton model* [4,5], both developed around the same time but independently, try to explain life as a functionally closed and self-sustaining chemical system. In other words, autopoietic systems and chemotons organize the production of their own components in such a way that these components are continuously regenerated and therefore maintain the chemical network processes that produce them. The notion of a *boundary* (such as a cell membrane) is essential in both of these models, physically separating the system from its environment, but allowing certain nutrients to enter and waste products to leave. However, this boundary layer must be produced by the system itself, and in turn promote the further production of its constituent components [3].

Even though these “metabolism-centered” models were already developed four decades ago, they never received much attention in a biological worldview that was (and still is) dominated by a focus on explicit, template-based, information storage and replication in nucleic acid polymers (DNA and RNA). However, with an increasing “systems” view in chemistry and biology, it is worth (re)considering these original models.

Autopoiesis and chemotons explain the workings of (cellular) life as it exists today. However, they do not necessarily explain how this kind of life came to exist in the first place, i.e., how an autopoietic system or chemoton emerges from basic (non-living) chemistry. Both models assume that the complete system and necessary processes are already present, and then show why and how they are self-sustaining. A more recent model, that of an *autogen* [6], tries to explain the actual spontaneous emergence of such a functionally-closed, self-sustaining system from pure chemistry. It does so by explicitly considering the (higher-order) *constraints* that the various parts of the system impose on each other (next to their mutual promotion). Here, too, the notion of a (self-generated) boundary is essential, both promoting and limiting the chemical reaction network that it encloses, in a synergistic and reciprocal way.

A more general and abstract model of a functionally closed, self-sustaining chemical reaction system is that of *collectively autocatalytic sets* [7-9]. Recently, the concept and analysis of autocatalytic sets has been developed more formally within so-called RAF (*R*eflexively *A*utocatalytic and *F*ood-generated) theory [10]. However, one element that is not explicitly represented in the formulation of autocatalytic sets and RAF theory is the notion of a boundary, an element that is not only explicit, but also essential in the other models mentioned above.

Here, we will show that the notion of a boundary can be easily incorporated within the formal RAF framework. Furthermore, by generalizing the notion of catalysis only slightly, this provides a direct mechanism for the emergence of higher-level autocatalytic (RAF) sets, and a necessary condition for their possible evolvability. This, therefore, could allow for a formal analysis (at least in part) of autopoietic systems, chemotons, and autogens within the RAF framework, enabling the application of its tools and results to these other model systems as well.

### Autocatalytic sets

First, we define a *chemical reaction system* (CRS) as a tuple $Q=\{X,\mathcal {R},C\}$ consisting of a set of molecule types *X*, a set of chemical reactions , and a catalysis set *C* indicating which molecule types catalyze which reactions. We also consider the notion of a food set *F*⊂*X*, which is a subset of molecule types (“nutrients”) that are assumed to be freely available from the environment. Informally, an *autocatalytic set* (or RAF set) is now defined as a subset $\mathcal {R}' \subseteq \mathcal {R}$ of reactions (and associated molecule types) which is:

1. *Reflexively Autocatalytic* (RA): each reaction $r \in \mathcal {R}'$ is catalyzed by at least one molecule type involved in $\mathcal {R}'$, and
2. *Food-generated* (F): all reactants in $\mathcal {R}'$ can be created from the food set *F* by using a series of reactions only from $\mathcal {R}'$ itself.

This definition captures the idea of life as a functionally closed (RA) and self-sustaining (F) chemical reaction network. A more formal (mathematical) definition of RAF sets is provided in [11-13], including an efficient (polynomial-time) algorithm for finding RAF sets in a general CRS, or determining that no such RAF exists. This RAF algorithm returns the unique *maximal* RAF (maxRAF) within a given CRS, or the empty set if the CRS does not contain any RAF set. It was shown that a maxRAF can often be decomposed into several smaller subsets which themselves are RAF sets (subRAFs) [14]. If such a subRAF cannot be reduced any further without losing the RAF property, it is referred to as an *irreducible* RAF (irrRAF) [12].

Some of the main findings of RAF theory are that autocatalytic sets are highly likely to exist in random (polymer-based) models of reaction networks once a critical level of catalysis is exceeded. This critical transition point already occurs at very modest levels of catalysis: between one and two reactions catalyzed per molecule type for moderate sized networks [12]. Moreover, only a linear growth rate in this critical level of catalysis is required to get RAF sets with high probability for increasing polymer lengths [12,15]. These results hold up under a variety of more realistic model extensions, and even for non-polymer systems [13,16-18]. Generally, there exist many hierarchical levels of subRAFs [14], which under appropriate conditions can give rise to the evolvability of autocatalytic sets [19]. Finally, the formal RAF framework can be directly applied to real chemical and biological systems to analyze the emergence and structure of autocatalytic sets [20,21].

### Boundaries in RAF sets

To show how the notion of a boundary can be incorporated into the formal RAF framework, and how this can give rise to the emergence of higher-level RAF sets, we provide a simple example that is partly inspired by a chemical system described in [6]. Our example system consists of the following reactions:

$$\begin{array}{lrll} r_{1}: & f_{1} + f_{2} & \rightarrow & a \\ r_{2}: & f_{2} + f_{3} & \rightarrow & b + c \\ r_{3}: & a + b & \rightarrow & d \\ r_{4}: & c & \rightarrow & e \\ r_{5}: & e + e^{n} & \rightarrow & e^{n+1} (n<L). \end{array} $$

In reaction *r*_5_, *e*^*n*^ denotes an “aggregate” of *n* “monomers” *e* bonded together into a macro-molecule. Thus, reaction *r*_5_ is really just shorthand for a family of *L*−1 reactions, each of which attaches the next monomer *e* to an already existing aggregate *e*^*n*^, making it one element larger (*e*^*n*+1^). This process starts by attaching two monomers *e* to produce the smallest possible aggregate *e*^2^ and builds aggregates up to a maximal size *L* (for technical reasons we impose a finite limit, but in practice this limit can be set arbitrarily high).

Next assume that *f*_1_, *f*_2_, and *f*_3_ are food molecules (nutrients) and that each of the reactions *r*_1_– *r*_5_ are catalyzed by one of the molecule types in the system. The full example CRS is defined as follows:

$$\begin{array}{rcl} X & = & \left\{f_{1}, f_{2}, f_{3}, a, b, c, d, e^{*}\right\} \\ \mathcal{R} & = & \left\{r_{1}, r_{2}, r_{3}, r_{4}, r_{5}\right\} \\ C & = & \left\{(d,r_{1}), (a,r_{2}), (c,r_{3}), (d,r_{4}), (c,r_{5})\right\} \\ F & = & \left\{f_{1}, f_{2}, f_{3}\right\} \end{array} $$

**Figure 1:**
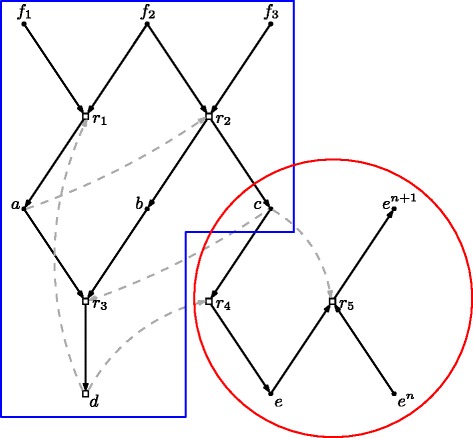
**The example CRS.** Black dots represent molecule types (the set *X*), white boxes represent reactions (the set ). Solid arrows going in an out of reactions represent reactants and products. Dashed gray arrows represent catalysis (the set *C*). The network as a whole is an RAF set for the food set *F*={*f*_1_,*f*_2_,*f*_3_}. The red and blue shapes contain subRAFs (see text).

In the definition of *X*, *e*^∗^ is again shorthand, this time for the set of *L* molecules {*e*,*e*^2^,⋯,*e*^*L*^}. A graphical (reaction network) representation of this CRS is shown in Figure 1 (the red and blue outlines will be explained shortly).

Note that this reaction network is mostly meant to illustrate the basic ideas discussed here, and does not represent any “real” system. However, RAF theory can, and has been, applied to real chemical networks, including an experimental RNA system [20] and the metabolic network of *E. coli* [21] (which was earlier shown to contain autocatalytic components [22]). Furthermore, the catalysts in this simple network are not necessarily fully evolved enzymes, but could for example be considered (organic or inorganic) cofactors, which presumably were the very first catalysts in the origin of life [21,23].

Given the food set *F*, this CRS forms a (maximal) RAF set consisting of all reactions in . Moreover, it contains an irreducible RAF set of three reactions, $\mathcal {R}_{1} = \left \{r_{1}, r_{2}, r_{3}\right \}$ (contained within the blue rectangle). Note that none of these RAF sets are immediately “constructible” (i.e., a CAF [15]). Some of the reactants of these reactions are in the food set *F*, but none of the catalysts are, so none of the reactions in  can proceed catalyzed initially. However, if reaction *r*_1_ were to happen spontaneously (uncatalyzed) at least once, which is always possible although at a lower rate, then the RAF set can come into existence: *r*_1_ creates the catalyst (*a*) for *r*_2_ and one of the reactants (*a*) for *r*_3_, *r*_2_ then creates the catalyst (*c*) and the other reactant (*b*) for *r*_3_, the reactant (*c*) for *r*_4_, and the catalyst (*c*) for *r*_5_, *r*_3_ subsequently creates the catalyst (*d*) for *r*_4_, and finally *r*_4_ creates the catalyst for *r*_1_ and the required monomers for *r*_5_.

Since the irrRAF $\mathcal {R}_{1}$ is itself an RAF set, it can exist without reactions *r*_4_ and *r*_5_. This irrRAF is roughly equivalent to a *viable core* in [19]. Reactions *r*_4_ and *r*_5_, on the other hand, are dependent on some of the reaction products (*c* and *d*) that are generated by $\mathcal {R}_{1}$, and thus do not form an RAF set by themselves. However, they can extend $\mathcal {R}_{1}$ to form a larger RAF. The subset $\mathcal {R}_{2} = \left \{r_{4}, r_{5}\right \}$ (contained within the red oval) is what is called a *co-RAF* in [24], or a *periphery* in [19].

Once the irrRAF $\mathcal {R}_{1}$ has come into existence (e.g. after a spontaneous occurrence of reaction *r*_1_), we could consider the closure $\text {cl}_{R_{1}}(F)$ of the food set *F* relative to the reaction set $\mathcal {R}_{1}$ to be an “extended” food set *F*^′^. The *closure* of a subset of molecules *X*^′^ relative to a subset of reactions $\mathcal {R}'$ is the set of all molecules that can be produced from *X*^′^ using only reactions from $\mathcal {R}'$ [12]. In this example, $F' = \text {cl}_{R_{1}}(F) = \left \{f_{1},f_{2},f_{3},a,b,c,d\right \}$. Now, relative to this extended food set *F*^′^, the subset $\mathcal {R}_{2}$*is* an RAF set. So, one RAF subset can create the right conditions for another RAF subset to come into existence (as already argued in [14]), in this case by generating an appropriate extended food set.

The products of $\mathcal {R}_{2}$ (the aggregates *e*^*n*^) do not directly interact with reactions in $\mathcal {R}_{1}$, neither as reactants nor as catalysts. However, suppose that once an aggregate *e*^*n*^ exceeds a certain size, say *B*≤*n*≤*L*, it can close in on itself (as with, e.g., lipid layers [25]) and form a boundary within which the irrRAF $\mathcal {R}_{1}$ can be contained. As a consequence, the rate at which the reactions in $\mathcal {R}_{1}$ happen will now be increased, simply by maintaining the relevant molecules (reactants and catalysts) in close proximity, instead of having them diffuse away into the environment.

**Figure 2:**
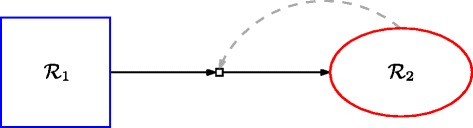
**The emergent RAF set.** $\mathcal {R}_{1}$ provides the food set *F* ^′^ (it is itself an RAF set for the original food set *F* and generates an extended food set *F* ^′^), and gives rise to (enables) $\mathcal {R}_{2}$. $\mathcal {R}_{2}$ then “catalyzes” its own production by forming a boundary within which the reactions in $\mathcal {R}_{1}$ can happen at increased rates.

Since the definition of a catalyst is a chemical element that increases the rate at which a reaction happens, without being used up in the reaction itself, the boundary can actually be considered an additional “catalyst” for the reactions in $\mathcal {R}_{1}$. In the example CRS given above, this would mean adding (*e*^*n*^,*r*_1_), (*e*^*n*^,*r*_2_), and (*e*^*n*^,*r*_3_), *B*≤*n*≤*L*, to the catalysis set *C*. More generally, the boundary can be considered as a catalyst for $\mathcal {R}_{1}$ as a whole. So, what we then have is two RAF subsets, $\mathcal {R}_{1}$ and $\mathcal {R}_{2}$, where the irrRAF $\mathcal {R}_{1}$ produces (*enables*) the co-RAF $\mathcal {R}_{2}$ by generating an extended food set, and $\mathcal {R}_{2}$ catalyzes its own production by speeding up the rate at which the reactions in $\mathcal {R}_{1}$ happen. In other words, an RAF (super)set of RAF (sub)sets, or a higher-level, emergent RAF set, as speculated earlier in [14]. This example of an emergent RAF is depicted in Figure 2.

Note that the boundary (*e*^*n*^) could also be considered as a catalyst for its own formation (reaction *r*_5_), as lipid layers usually enable the incorporation of further lipids. However, we have not explicitly included this in our example, as it does not make a direct difference for the main ideas discussed here (i.e., the emergence of higher-level RAFs).

In conclusion, the notion of a boundary can be incorporated into the RAF framework by extending the notion of catalysis slightly: considering a boundary as an (additional) catalyst for the reactions that happen within its enclosure. This immediately gives rise to a mechanism for the emergence of higher-level RAF sets, and for their possible evolvability. In [19] it was shown that two necessary conditions for evolvability of autocatalytic sets are (1) having a large enough number of “viable cores” (irreducible RAF sets) (2) existing in various combinations within compartments. In [14] we already showed that, in principle, there can be exponentially many irrRAFs within a given (max)RAF. Here we have shown how boundaries (compartments) can also be incorporated within the RAF formalism.

## Conclusions

The above example of how boundaries can be incorporated within the formal RAF framework shows how this essential element in other models of functionally closed, self-sustaining systems can be represented and analyzed in the context of RAF sets. Furthermore, the chemoton model has two complementary (autocatalytic) reaction networks within such a self-generated boundary (“membrane system”): a metabolic network (“cyclic subsystem”) and an informational network (“genetic subsystem”) [4,5]. In [17], a partitioned polymer model was studied in the context of RAF sets where reactions can only involve molecule types from one of two partitions (e.g., either only RNA or only peptides), but catalysis can be both within and across partitions. This study showed that the existence of RAF sets is equally likely (and for similar levels of catalysis) as in a standard non-partitioned polymer model. Thus, systems with an explicit distinction between a metabolic and a genetic network can also be dealt with in terms of RAF sets. Finally, to model a possibly semi-permeable boundary, additional “transport” reactions can be included in the CRS that indicate which molecule types can cross the boundary in one or both ways.

Whether *all* aspects of these other models can be fully captured within the RAF framework seems a more difficult question. Gánti, in the context of his chemoton model, talks about “constrained chemical paths” in the metabolic subsystem, which is (at least partly) controlled by the genetic subsystem [5]. Constraints, imposed by the system’s own structure and functionality, are also an essential aspect in the autogen model [6]. However, the notion of constraints is (currently) not formalized in the RAF framework. For example, a boundary can act both as a promotor (catalyst) by keeping the relevant molecules in close enough proximity so that they can actually react, as well as a constraint (inhibitor) by preventing some of the relevant molecules (nutrients) from entering the system. It is known that including inhibition in a CRS makes the general problem of finding RAF sets NP-complete [15], although recent developments show that the problem is still tractable if the total number of inhibitors is limited [26]. But whether including inhibition (an important factor in biological regulation) is sufficient to formally capture the notion of constraints, remains to be explored further.

Note that the reverse direction is not necessarily true: not every RAF set is an autopoietic system, chemoton, or autogen. In fact, whereas RAF theory is mostly a *descriptive* framework that can be used to represent and analyze a given (known) reaction network, the other models actually try to provide a *mechanistic* account of how self-generation, self-sustainability, and self-regulation can exist or even spontaneously emerge in purely chemical systems. However, the RAF framework seems able to represent these various models in a formal way at least to a significant extent, and could thereby serve as the basis for a useful analysis tool and unifying formal framework, contributing to the further development and study of such models. Furthermore, since RAF theory does not explicitly require the notion of a boundary, it can also be used to model and analyze chemical networks that are possibly relevant to the origin of life but which do not (yet) create their own boundary, such as in hydrothermal vents in naturally occurring micropores [27] or on “catalytic” surfaces [28].

In this brief perspective we have attempted to “delve deeper into the comparison between these three views (Maturana and Varela, Gánti, and Kauffman)” [3], also including the more recent view of Deacon [6]. We hope that this comparison will help in a constructive way towards a full convergence of these various views, models, and methods.
